# Checkpoint inhibitors, fertility, pregnancy, and sexual life: a systematic review

**DOI:** 10.1016/j.esmoop.2021.100276

**Published:** 2021-09-28

**Authors:** M. Garutti, M. Lambertini, F. Puglisi

**Affiliations:** 1 CRO Aviano National Cancer Institute IRCCS, Aviano, Italy; 2 Department of Medical Oncology, Breast Unit, IRCCS Ospedale Policlinico San Martino, Genova, Italy; 3 Department of Internal Medicine and Medical Specialties (DiMI), School of Medicine, University of Genova, Genova, Italy; 4 Department of Medicine (DAME), University of Udine, Udine, Italy

**Keywords:** fertility, immunotherapy, checkpoint inhibitors, sexuality, pregnancy

## Abstract

Immune checkpoint inhibitors (i.e. anti-PD1, anti-PDL1, and anti-CTLA4) have revolutionized the therapeutic approach of several cancer types. In a subset of metastatic patients, the duration of the response is so long that a cure might be hypothesized, and a treatment discontinuation strategy could be proposed. Considering that long-term efficacy, some patients could also plan to have a child. Moreover, immunotherapy is moving to the early setting in several diseases including melanoma and breast cancer that are common cancers in young patients. However, there is a paucity of data about their potential detrimental effect on fertility, pregnancy, or sexuality. Herein, we conducted a systematic review with the aim to comprehensively collect the available evidence about fertility, pregnancy, and sexual adverse effects of checkpoint inhibitors in order to help clinicians in daily practice and trialists to develop future studies.

## Highlights

• This review collected the available evidence on the role of immunotherapy on fertility, hypogonadism, and sexuality.
• Immune checkpoint inhibitors could impair fertility.
• Immunotherapy can cause primary hypogonadism and secondary hypogonadism.
• Immune checkpoint inhibitors have to be avoided during or shortly before pregnancy.
• Immunotherapy might alter sexuality and libido.

## Introduction

Immune checkpoint inhibitors (ICIs; i.e. anti-PD1, anti-PDL1, and anti-CTLA4) have revolutionized the therapeutic landscape in oncology.^1^, ^2^, ^3^ In particular, these compounds have increased the survival in both the metastatic and adjuvant settings in several types of malignancies.^1^, ^2^, ^3^ In a subset of metastatic patients, the duration of the response is so long that a cure might be hypothesized, and a treatment discontinuation strategy could be proposed.^4^, ^5^, ^6^, ^7^, ^8^, ^9^ In light of the long-term efficacy, some patients could also plan to have a child. Moreover, immunotherapy is moving to the early setting in several diseases including melanoma and breast cancer that are common cancers in young patients.^10^, ^11^, ^12^

In opposition to the vast body of evidence regarding the clinical utility of ICIs, there is a paucity of data about any detrimental effect on fertility, future pregnancies, or sexuality.^13^ This gap of knowledge could complicate the therapy proposal, especially in young patients. This is of particular importance in light of the European Society for Medical Oncology (ESMO) and European Society of Human Reproduction and Embryology guidelines recommending a fertility counseling in all patients, including those in the metastatic setting.^14^^,^^15^ Therefore, the unknown gonad toxicity of immunotherapy represents an important unmet need in this field.

Herein, we conducted a systematic review (see Supplementary Appendix S1, available at https://doi.org/10.1016/j.esmoop.2021.100276) with the aim to comprehensively collect the available evidence about fertility, pregnancy, and sexual adverse effects of ICIs in order to help clinicians in daily practice and researchers to develop future studies. In particular, we describe four major classes of adverse effects: primary hypogonadism, secondary hypogonadism, pregnancy impairment, and altered libido and sexual life. Finally, we discuss some practical clinical issues linked to fertility and sexuality and a possible methodology for future clinical trials.

## Primary hypogonadism

Primary hypogonadism refers to the direct damage of gonads, that is, ovaries or testicles.^16^^,^^17^ Clinically, this translates into a reduced or impaired production of viable oocytes or spermatozoa and a fertility compromise. From a biochemical perspective, primary hypogonadism can be suspected by a reduced level of sexual hormones (e.g. testosterone and estradiol) with a concomitant increase of gonadotropins [follicle-stimulating hormone (FSH) and luteinizing hormone (LH)].^18^^,^^19^ In women, there could also be a reduction of anti-Müllerian hormone concentration,^18^^,^^19^ a substance that has been linked to the ovarian reserve and, therefore, with the reproductive potential.^20^

Some data show that ICIs might cause primary hypogonadism (Table 1). However, the evidence is weak and in the form of case reports or case series. Moreover, to the best of our knowledge, no data in women have been published.

**Table 1:** Evidence of checkpoint inhibitors on primary hypogonadism, secondary hypogonadism, and sexuality

PMID	Adverse effect	Evidence	Drug	Sample size
28039179	Primary hypogonadism	Bilateral orchitis	Anti-PD1 + anti-CTLA4	1
30936376	Primary hypogonadism	Epididymo-orchitis + encephalitis	Anti-PD1	1
32556068	Primary hypogonadism	Altered spermatogenesis	Anti-PD1 + anti-CTLA4	7
33613847	Primary hypogonadism	Testosterone deficiency	Anti-PD1 and/or anti-CTLA4	49
33299797	Primary hypogonadism	Azoospermia	Anti-PD1 + anti-CTLA4	1
31235040	Primary hypogonadism	Azoospermia	Anti-PD1 and/or anti-CTLA4	22
30861560	Secondary hypogonadism	Metanalysis of endocrine irAEs	Anti-PD1 and/or anti-CTLA4	19922
31021376	Secondary hypogonadism	Metanalysis of irAEs	Anti-PD1 or anti-PDL1	20128
32507965	Secondary hypogonadism	Endocrine irAEs	Anti-PD1 and/or anti-CTLA4	6089
24610577	Secondary hypogonadism	Endocrine irAEs	Anti-CTLA4	256
31235040	Secondary hypogonadism	Isolated hypogonadotropic hypogonadism	Anti-PDL1	1
33646368	Secondary hypogonadism	Increased LH-to-FSH ratio and estradiol	Anti-PD1	22
31235040	Sexuality	No sexual alterations during or after ICIs	Anti-PD1 and/or anti-CTLA4	25
31672171	Sexuality	Autoimmune vulvitis	Anti-PD1	1

FSH, follicle-stimulating hormone; ICI, immune checkpoint inhibitor; irAES, immune-related adverse events; LH, luteinizing hormone.

Two case reports described a case of orchitis and epididymal-orchitis during treatment with anti-PD1/anti-CTLA4 and anti-PD1, respectively.^21^^,^^22^ In the first case, there was a spontaneous resolution of the orchitis, while the second one also developed encephalitis, leading to a steroid therapy initiation with subsequent regression of symptoms.

In another case report, a normozoospermic man treated with anti-PD1 and anti-CTLA4 developed azoospermia 2 years after the immunotherapy.^23^

In a recent case series, Scovell et al.^24^ retrospectively analyzed testicular histology of patients treated with anti-PD1 and anti-CTLA4 that underwent autopsy. An age-matched control cohort not treated with immunotherapy was used. Of the seven men treated with ICIs, six (86%) had impaired spermatogenesis, including one focal active spermatogenesis, two hypospermatogenesis, and three Sertoli-cell-only syndrome. In the control group, only two of the six men (33%) had impaired spermatogenesis.

In a recent cross-sectional pilot study, Salzmann et al.^25^ analyzed the sperm of 22 patients currently or previously treated with ICIs. Among them, 82% had a normal spermiogram, three showed azoospermia, and one oligoasthenoteratozoospermia. However, three patients with pathologic spermiogram had significant confounding factors (previous inguinal radiotherapy, chemotherapy and chronic alcohol abuse, and bacterial orchitis). On the contrary, one patient with a normal pretreatment spermiogram showed azoospermia with an inflammatory infiltrate after ICIs therapy. While these data suggest that primary hypogonadism in male might be a rare immune-related adverse effect, it should be noted that only five patients in this study had a pretreatment spermiogram available, making it difficult to draw any definitive conclusion.

Finally, the retrospective study of Peters et al.^26^ showed that low testosterone levels were present in 34 of 49 (69%) men treated with anti-PD1 and/or anti-CTLA4. Interestingly, the vast majority of patients reported fatigue, but only three were treated with testosterone replacement therapy. Although this study is speculative, several methodological biases limit the demonstration of causality between checkpoint inhibitors and the drop in testosterone levels. For example, only 61% of patients had a baseline testosterone measurement, and the sampling time during the treatment was inconstant. However, from a preclinical perspective, an alteration of testicles during immunotherapy seems to be possible.^27^ Indeed, it has been shown that monkey testicular weight decreases during treatment with anti-CTLA4, even though sperm did not show any histopathological changes. Moreover, anti-CTLA4 seems to bind ovarian connective tissue in monkeys, but no histopathologic changes have been documented.^27^

Comprehensively, ICIs might cause primary hypogonadism, especially in men. However, the frequency of this adverse effect, its magnitude, the duration after the discontinuation of immunotherapy, and the implications on fertility are unknown.

## Secondary hypogonadism

Secondary hypogonadism refers to the damage in the hypothalamus or, more frequently, in the pituitary gland causing a reduced activation of the hypothalamic–pituitary–gonadal axis.^28^^,^^29^ Clinically, this translates into a reduced or impaired production of viable oocytes or spermatozoa and a compromised fertility.^28^^,^^29^ Biochemically, secondary hypogonadism can be suspected when reduced levels of sexual hormones (e.g. testosterone and estradiol) coexist with a concomitant decrease of gonadotropins (FSH and LH).^28^^,^^29^ Of note, secondary hypogonadism often arises in panhypopituitarism, which also causes secondary hypothyroidism and secondary adrenal insufficiency.

Hypophysitis and panhypopituitarism are well-known and well-described adverse events of checkpoint inhibitors (Table 1). However, most data focus on consequential adrenal insufficiency and hypothyroidism because of their potential life-threatening consequences. Nevertheless, in the context of putative curable disease or long-lasting remission, the reproductive sequelae should be considered.

Anti-PD1, anti-CTLA4, and their combinations can cause hypophysitis, but with different frequencies.^30^ The rate of hypophysitis is 5.6% for anti-CTLA4, 0.5%-1.1% for anti-PD1, and 8.8-10% for the combination.^30^ Moreover, the rate of grade 3-4 toxicities seems to be higher for anti-PD1 compared with anti-PDL1.^31^^,^^32^ Of note, endocrine toxicities, including hypophysitis, seem to be a chronic adverse effect.^33^^,^^34^ In comparison to hypophysitis, hypogonadism is described as an uncommon adverse event.^31^^,^^35^ However, it is unknown how many hypogonadisms are primary or secondary, and an underestimation of hypogonadism seems to be plausible in light of the absence of routine testing of sex hormones.^31^^,^^35^ The lack of widespread biochemical testing of FSH, LH, testosterone, and estradiol might be relevant because a report showed that hypogonadotropic hypogonadism could manifest even in the absence of alteration of other endocrine axes (e.g. pituitary–thyroid and pituitary–adrenal).^36^ In other words, secondary hypogonadism may arise without panhypopituitarism, making its diagnosis challenging. However, the frequency of isolated hypogonadotropic hypogonadism during treatment with checkpoint inhibitors remains unknown and needs to be clarified.

Finally, as shown by Tulchiner et al.,^37^ patients treated with anti-PD1 could develop an increased LH-to-FSH ratio and estradiol levels. However, for an unknown reasons, this phenomenon seems to occur only in men.

Comprehensively, hypopituitarism is a well-demonstrated side-effect of ICIs. However, more information needs to be collected on the frequency and the magnitude of disruption of the pituitary–gonadal axis by these compounds. In addition, the effect of immunotherapy-induced hypopituitarism on fertility remains unknown.

## Pregnancy

Mother and fetus have a different genetic makeup. Therefore, an immunologic tolerance toward the father antigens has to be developed to avoid miscarriage. This tolerance appears to be modulated by a myriad of molecular systems.^38^ While the complete coverage of such tolerogenic mechanisms is beyond the scope of this review, some of them will be briefly discussed (see Supplementary Table S1, available at https://doi.org/10.1016/j.esmoop.2021.100276 for a list of preclinical studies on PD1/PDL1/CTAL4). In particular, both PDL1 and CTLA4 are expressed at the fetomaternal interface during gestation and have a significant role in fetomaternal tolerance.^39^, ^40^, ^41^ Therefore a fetotoxic effect of anti-PD1/-PDL1 and anti-CTLA4 could be anticipated.

The pivotal role of the PD1–PDL1 axis has been experimentally validated through the pharmacological inhibition of PDL1 in a model of allogeneic mice pregnancy.^40^^,^^42^^,^^43^ Upon blocking this axis, there is a fivefold increase in the risk of miscarriage.^40^ However, this effect was seen only in allogeneic pregnancy and not in the syngeneic one. This observation highlights that fetomaternal immunotolerance and PDL1 expression are modulated by the degree of allogenicity (the more the allogenicity, the more important are the immune tolerance mechanisms). Therefore the effects of anti-PD1/anti-PDL1 antibody on the fetus are anticipated to be patient specific and strongly linked to the paternal antigenic components.

Besides, the role of CTLA4 was experimentally validated. Indeed, in a monkey model, anti-CTLA4 treatment caused reduced maternal weight, higher abortion rates, stillbirth, and premature delivery.^44^ These adverse effects were seen mainly in the third trimester of pregnancy.

Unlike many chemotherapeutic agents that exert fetotoxic effects in the first trimester of pregnancy,^45^^,^^46^ ICIs might have the maximum toxicity in the third.^38^^,^^47^ Although validated data of trimester-specific toxicity are lacking, it is well-known that the placenta changes its capacity to transport immunoglobulins during months.^48^^,^^49^ In particular, placental immunoglobulin Gs (IgGs) are relatively scarce during the first 6 months, while, in the last trimesters, they sharply increase, reaching similar or superior levels to those of maternal blood.^48^^,^^49^ Moreover, it appears that IgG subclasses impact antibody transport across the placenta. IgG1s are the globulins transported with the best efficiency, followed by IgG4, IgG3, and IgG2.^48^, ^49^, ^50^, ^51^ Table 2 lists the available checkpoint inhibitors classified by their theoretical capacity to cross the placenta.

**Table 2:** Checkpoint inhibitors and their capacity to cross the placenta

Drug	Target	IgG subtype	Placenta permeability
Atezolizumab	PDL1	IgG1	++++
Avelumab	PDL1		
Durvalumab	PDL1		
Ipilimumab	CTLA4		
Zalifrelimab	CTLA4		
Cemiplimab	PD1	IgG4	+++
Nivolumab	PD1		
Pembrolizumab	PD1		
Tislelizumab	PD1		
Tremelimumab	CTLA4	IgG2	+

IgG, immunoglobulin G.

From a clinical point of view, there is a paucity of data on the fetotoxic effect of anti-PD1/anti-PDL1/anti-CTLA4. This could be explained by the recommendation to avoid these compounds during pregnancy based on preclinical evidence discussed above. However, in the last years, some case reports have been published^52^, ^53^, ^54^, ^55^, ^56^, ^57^ (Table 3). Thus, it appears that, at least in some cases, pregnancy and childbirth are compatible with anti-PD1 and/or anti-CTLA4 therapy. However, it is not possible to interpolate the real frequency of regular pregnancy/delivery. Nevertheless, while case reports could suffer the positive-result bias,^58^ an unpublished communication (D. Minor, January 2017^52^) described seven cases of women present in the Food and Drug Administration (FDA) database that were treated with anti-CTLA4 during pregnancy. Among them, there was a case of spontaneous abortion, one stillbirth, one ectopic pregnancy, two pregnancies terminated by voluntary abortion, and two pregnancies with unknown outcomes.^52^ No information on malformation or immune-mediated fetal adverse events was present on the FDA database. From the six case reports available, none of the fetuses experienced a malformation linked to immunotherapy, and only one had potentially immune-mediated hypothyroidism (Table 3).

**Table 3:** Clinical evidence of checkpoint inhibitors usage during pregnancy

PMID	Size	Drug	Trimester	Successful pregnancy	Fetal irAEs	Fetal outcomes
30241195	1	Anti-CTLA4 + intratumoral anti-IL2	I	Yes	No	Normal development milestones at 2/3/4 years
30241195	7	Anti-CTLA4	NA	0-2^a^/7	NA	NA
30262400	1	Anti-CTLA4 + anti-PD1	I	Yes	No	Normal development milestones at 11 months
30454709	1	Anti-CTLA4 + anti-PD1	II	Yes (preterm)	No	Normal development milestones at 6 months compatible with preterm delivery
33768686	1	Anti-PD1	I	2 (twins)	No	Normal development milestones at 9 months (one twin was missing the left hand, which was interpreted as a consequence of strangulation by amniotic cord)
30730328	1	Anti-PD1	I	1 (preterm)	Yes (thyroiditis)	Normal development milestones at 6 months compatibly with preterm delivery
32073510	1	Anti-CTLA4 + anti-PD1	I	2 (twins, preterm)	No	Discharged from neonatal care intensive unit after 28-30 days

irAEs, immune-related adverse events; NA, not available.

a One spontaneous abortion, one stillbirth, one ectopic pregnancy, two pregnancies terminated by voluntary abortion, and two pregnancies with unknown outcomes.

Comprehensively, while checkpoint inhibitors seem to be fetotoxic, this adverse effect might be patient specific, varying by the antigenicity of the fetus. Nevertheless, in some patients, normal pregnancy and delivery seem to be possible even though the abortion-to-delivery ratio in a general population remains unknown. Moreover, the impact of immunotherapy on future pregnancies and the presence of checkpoint inhibitors in breast milk have to be clarified.

## Libido and sexual life

Although many phase III clinical trials with ICIs evaluated the quality of life as a secondary endpoint, sexuality remains a neglected topic (Table 1). To the best of our knowledge, only two studies evaluated this topic.

The first study is a case report that describes the development of vulvitis in a woman treated with an anti-PD1.^59^ Although autoimmune phenomena to external genitalia could severely impair sexuality, the exact incidence of these phenomena is currently unknown.

The second is a pilot cross-sectional study involving 25 males currently or previously treated with ICIs. None of them reported an impairment of sexual function or sexual activity. Only one reported a light restriction of erectile function.^25^ While these data seem to suggest a limited toxicity of ICIs on sexuality, a larger sample size and a prospective design are needed to draw any firm conclusions.

Despite the current gap of knowledge, a special consideration regarding hypophysitis and its consequences on sexuality can be made. Impairment of the pituitary–gonadal axis might culminate in a reduction of sexual hormones. It is well known that sexual hormonal deficiency can reduce fertility and lead to physical– and psychological–sexuality disturbance.^60^^,^^61^

## Current clinical practices

Despite the exiguity of data regarding fertility and sexuality perturbations by checkpoint inhibitors, some pragmatic approaches applicable in daily clinical practice can be depicted (Table 4).

**Table 4:** Pragmatic clinical approach and unsolved questions on checkpoint inhibitors, fertility, and sexuality

Adverse event	Clinical approaches	Unsolved questions
Primary hypogonadism	• Discuss the possibility of infertility linked with the treatment and the strategies of fertility preservation (e.g. cryopreservation)	• Frequency • Duration after discontinuation of immunotherapy • Alteration of sex hormones levels (e.g. testosterone, estradiol) • Impact on fertility, pregnancy, and complicated pregnancy • Impact on libido and sexual life
Secondary hypogonadism	• Discuss the possibility of hypopituitarism-induced infertility linked with the treatment and the strategies of fertility restoration (e.g. hormonal therapy if clinically safe)	• Frequency of secondary hypogonadism • Frequency of isolated secondary hypogonadism • Impact on fertility, pregnancy, and complicated pregnancy • Impact on libido and sexual life
Pregnancy	• Strongly suggest avoiding pregnancy during treatment using at least one contraceptive methods • In case of pregnant woman, discuss the current evidence and risks of concurrent immunotherapy administration. Discuss the possibility of treatment discontinuation in case of long-term complete response • If therapy will be administered during pregnancy, after delivery, follow-up the child for development abnormalities, autoimmune diseases • Strongly suggest a minimum time from the end of therapy and the beginning of a pregnancy (3 months for ipilimumab and durvalumab, 4 months for pembrolizumab, and 5 months for nivolumab and atezolizumab)	• Trimester-specific toxicity • Frequency of miscarriage, stillbirth, premature delivery • Frequency of fetal malformation, fetal autoimmune disease • Frequency of complicated pregnancy
Libido and sexual life	• Discuss the theoretical possibility of reduced libido and impaired sexual life • Consider hormone replacement therapy in case of deficiencies and if clinically indicated	• Frequency • Duration after discontinuation of immunotherapy • Type of disturbance • Impact on procreation • Impact on quality of life

First, regarding primary hypogonadism, it is essential to discuss with the patient the possibility of altered gametogenesis and the subsequent reduced fertility. Accordingly, fertility-preservation strategies^62^, ^63^, ^64^ (gamete cryopreservation) should be offered, especially in the curative setting where a cure can be achieved and family planning can be made. Although such a strategy could be pursued in a metastatic setting, it is better to avoid a delayed therapy initiation in favor of a fertility-preservation strategy, especially in high-burden disease. In addition, ovarian function preservation with luteinizing hormone-releasing hormone (LHRH) agonists, as used in other cancers^65^ (e.g. breast cancers^66^), should not be offered during treatment with immunotherapy given without cytotoxic therapy because of a lack of evidence on immunotherapy-related gonadotoxicity risk and the benefit of LHRH agonists in this setting. However, if ICIs are used together with chemotherapy, this strategy should be considered.^65^

Second, it is important to discuss the possibility of hypopituitarism-induced infertility and its virtual chronic persistence. However, even with hypopituitarism, it should be noted that it is possible to produce viable gametes and become pregnant, especially with adequate hormonal stimulations,^67^^,^^68^ but again, data on pregnancy rate after immunotherapy-induced hypopituitarism are not available.

Third, it should be recommended to avoid pregnancy during checkpoint inhibitor treatment. In particular, it is suggested to use at least one contraceptive method. If the woman gets pregnant or is already pregnant, it should be discussed along with the pros and cons of concurrent treatment. In the case of metastatic disease with a long-term complete response, treatment discontinuation could be discussed. After delivery, it is pivotal to follow-up child for potential complications including development of an autoimmune disease. As stated by others,^69^ prematurity has to be avoided as much as possible. While there are not high-quality data on the impact of immunotherapy on future pregnancy outcomes or the presence of checkpoint inhibitors in breast milk, a pragmatic approach could be to wait several months from the end of the treatment to the beginning of pregnancy or breastfeeding. In particular, as stated by the European Medicines Agency (EMA) and FDA technical sheets, the minimum time from the end of therapy should be 3 months for ipilimumab^70^ and durvalumab,^71^ 4 months for pembrolizumab,^72^ and 5 months for nivolumab^73^ and atezolizumab.^74^

Fourth, the virtual possibility of reduced libido and impaired sexuality, especially in the case of hypophysitis, should also be discussed. In such cases, an evaluation of the pituitary–gonadal axis might be helpful (e.g. FSH, LH, testosterone, estradiol). If sex hormone deficiency is diagnosed, a hormone replacement therapy should be considered, if clinically applicable. Of note, fatigue is a valuable and often underrated symptom of hypogonadism.^26^^,^^75^^,^^76^

## Future clinical trials

Considering the paucity of data regarding the effect of checkpoint inhibitors on fertility, pregnancy, and sexuality, there is an urgent need for new evidence to orientate clinical practice (Table 4). Although a retrospective study could be helpful and a precious starting point, a well-designed prospective trial could be the best solution. In particular, reproductive health outcomes should be included in standard toxicity assessments of all clinical trials.^77^

It seems that checkpoint inhibitors may cause primary hypogonadism, especially in men. However, the frequency of this side-effect needs to be clarified, as well as its duration after treatment discontinuation, the laboratory perturbations of sex hormones (e.g. testosterone, estradiol), and the impact on fertility, pregnancy, and regular delivery rate.

Regarding secondary hypogonadism, currently, there is strong evidence about the causative role of immunotherapy in hypophysitis and hypopituitarism. However, beyond the monitoring of thyroid and adrenal functions, better documentation of a pituitary–gonadal axis impairment is highly warranted. Moreover, because a case of isolated secondary hypogonadism has been described, documentation about its frequency in a real-world setting should be generated. Therefore the evaluation of FSH, LH, testosterone, and estradiol should be performed. Again, the implication on fertility and pregnancy of secondary hypogonadism needs to be evaluated.

While many fetotoxic effects of ICIs have been documented, the vast majority of evidence is preclinical. Therefore, a multi-institutional effort finalized to collect anecdotal data in humans accidentally exposed to these agents during pregnancy is highly warranted. Moreover, it is important to annotate the potential short- and long-term toxicities in children exposed *in utero* to these agents, including the risk of autoimmune diseases.

Finally, the consequences of ICIs on libido and sexuality are currently neglected. Therefore, the use of validated questionnaires^78^^,^^79^ at different timepoints could be carried out.

## Conclusions

ICIs have revolutionized cancer treatments because of their extraordinary efficacy. Therefore, it is anticipated that their use is going to increase further in the near future. Paradoxically, the toxicities induced by ICIs on fertility, pregnancy, and sexuality are poorly understood. From the currently available evidence, these compounds could cause primary hypogonadism, secondary hypogonadism, and, theoretically, libido and sexual impairment. In addition, based on preclinical data, conception and pregnancy should be avoided during treatment with anti-PD1/anti-PDL1/anti-CTLA4. Nevertheless, at least in some cases, a regular delivery seems to be possible.

The data discussed above can be helpful to clinicians to better aid patients in daily clinical practice. An international effort to bridge the current knowledge gap will be fundamental.
