# Corrigendum to ‘Checkpoint inhibitors, fertility, pregnancy, and sexual life: a systematic review’ [ESMO Open Volume 6, Issue 5, October 2021, 100276]

**DOI:** 10.1016/j.esmoop.2021.100291

**Published:** 2021-10-13

**Authors:** M. Garutti, M. Lambertini, F. Puglisi

**Affiliations:** 1 CRO Aviano National Cancer Institute IRCCS, Aviano, Italy; 2 Department of Medical Oncology, Breast Unit, IRCCS Ospedale Policlinico San Martino, Genova, Italy; 3 Department of Internal Medicine and Medical Specialties (DiMI), School of Medicine, University of Genova, Genova, Italy; 4 Department of Medicine (DAME), University of Udine, Udine, Italy

The authors regret that the affiliations for Professor F. Puglisi are incorrect. They should be:

CRO Aviano National Cancer Institute IRCCS, Aviano, Italy and Department of Medicine (DAME), University of Udine, Udine, Italy

The authors would like to apologise for any inconvenience caused.

