# Supplementary material for: Checkpoint inhibitors, fertility, pregnancy, and sexual life: a systematic review
Source: ESMO Open. 2021 Sep 28;6(5):100276. doi: 10.1016/j.esmoop.2021.100276 (PMC8487000; doi:10.1016/j.esmoop.2021.100276)
Supplement: Supplementary Appendix S1 [file mmc2.docx]

**Searching method**

A systematic research of literature was performed to find relevant papers focused on reproductive and sexual toxicities from checkpoint inhibitors published until the date of 11 July 2021. The research was performed by first author (MG) on PubMed, ASCO library, and ESMO library. The following research queries was used:

PubMed:

*(Prolgolimab OR “BCD 100” OR “BCD-100” OR Pembrolizumab OR Keytruda OR Lambrolizumab OR “Merck 3475” OR “MK 3475” OR “MK-3475” OR MK3475 OR “Sch 900475” OR “SCH-900475” OR Nivolumab OR Opdivo OR “BMS 936558” OR “BMS-936558” OR “MDX 1106” OR “MDX-1106” OR “ONO 4538” OR “ONO-4538” OR Ipilimumab OR Yervoy OR “BMS-734016” OR “MDX-010” OR “MDX-101” OR “MDX-CTLA-4” OR Atezolizumab OR Tecentriq OR “MPDL 3280A” OR “MPDL-3280A” OR “MPDL3280A” OR “RG-7446” OR “RG7446” OR Spartalizumab OR PDR001 OR Durvalumab OR Imfinzi OR “MEDI 4736” OR “MEDI-4736” OR MEDI4736 OR Cemiplimab OR Libtayo OR “Cemiplimab-rwlc” OR REGN2810 OR Camrelizumab OR “SHR-1210” OR “SHR1210” OR Sintilimab OR IBI308 OR Tislelizumab OR “BGB-A317” OR Toripalimab OR “JS 001” OR “JS-001” OR JS001 OR Dostarlimab OR “TSR 042” OR “TSR-042” OR Avelumab OR Bavencio OR Tremelimumab OR Ticilimumab OR “CP 675,206” OR “CP-675” OR “CP-675,206” OR “CP-675206” OR “anti PD1” OR “anti-PD1” OR “anti PD-1” OR “anti-PD-1” OR “anti CTLA4” OR “anti-CTLA4” OR “anti-CTLA-4” OR “anti CTLA-4” OR “anti PDL1” OR “anti-PDL1” OR “anti PD-L1” OR “anti-PD-L1” OR “anti-PDL-1” OR “anti PDL-1” OR “checkpoint inhibitor” OR “checkpoint inhibitors”) AND (miscarriage OR pregnancy OR childbearing OR gestation OR sperm OR “sperm count” OR “sperm counting” OR “sperm motility” OR fertility OR infertility OR sterility OR “feeding time” OR libido OR erectile OR “erectile function” OR orgasm OR sexual OR sex OR spermatogenesis OR gametogenesis OR oogenesis OR oophoritis OR “autoimmune ovarian failure” OR “autoimmune premature ovarian failure” OR orchitis OR “anti-müllerian hormone” OR “anti müllerian hormone” OR “anti mullerian hormone” OR “anti-mullerian hormone” OR “AMH” OR menopause OR abortion OR dispareunia OR testosterone OR estradiol OR oestradiol OR estrogen OR oestrogen OR hypogonadism OR teratogen OR teratogenicity OR teratogenic OR azoospermia OR hypospermia OR fetus OR foetus OR ((hypophysitis OR endocrine) AND (“systematic review” OR “meta-analysis” OR “meta analysis”)))*

ASCO library:

*(immuno OR checkpoint OR nivolumab OR pembrolizumab OR ipilimumab OR atezolizumab OR PD1 OR PD-1 OR PD-L1 OR PDL1 OR PDL-1 OR CTLA4 OR CTLA-4) AND (pregnancy OR sperm OR fertility OR infertility OR sterility OR libido OR orgasm OR sexual OR hypogonadism)*

ESMO library:

*pregnancy OR sperm OR fertility OR infertility OR sterility OR libido OR orgasm OR sexual OR hypogonadism*

**Inclusion criteria**

Papers were included if they are written in English, contained relevant clinical or preclinical data regarding fertility- or sexual-impairment caused by checkpoint inhibitors. Reviews could be considered if particularly relevant or because of original data.

**Flow diagram of the study selection process**


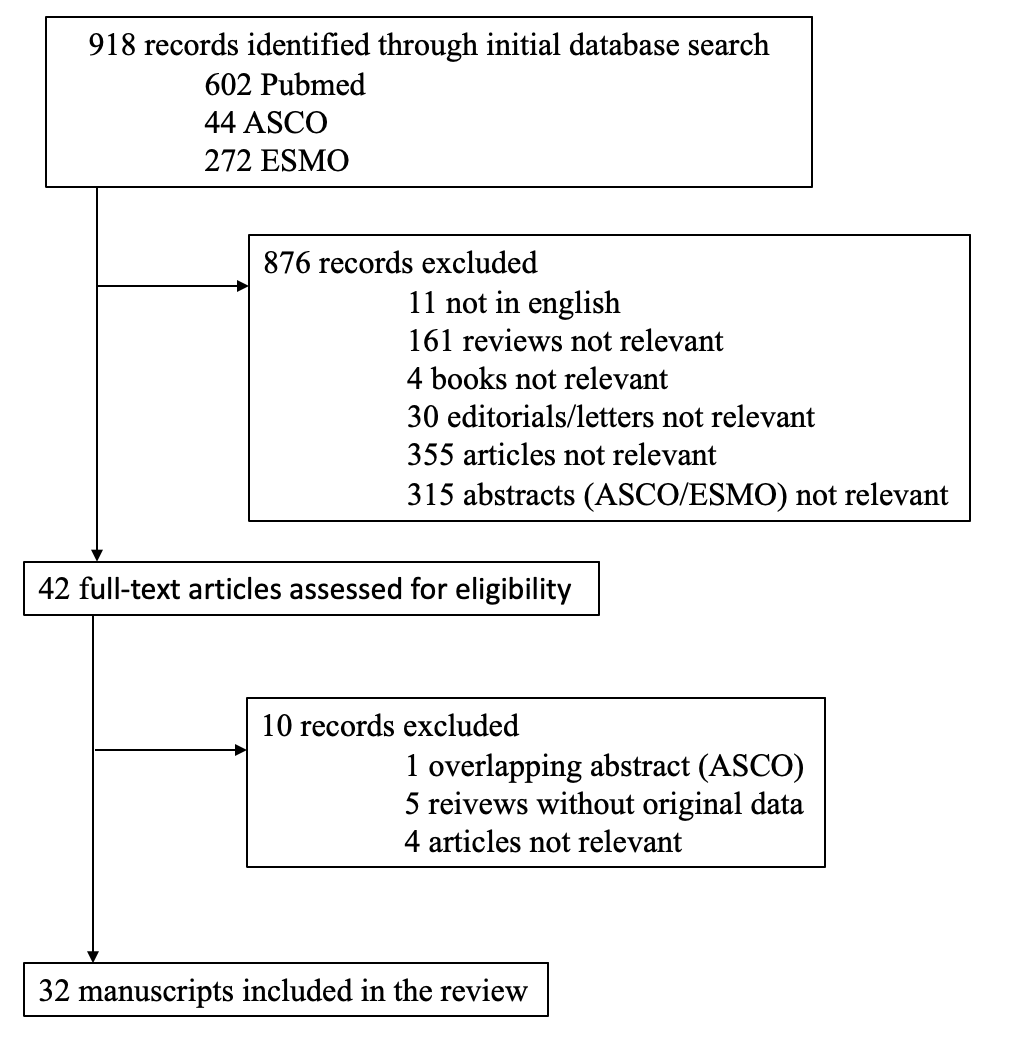


**List of included studies**

| **PMID** | **Topic** | **Setting** |
| --- | --- | --- |
| 33299797 | Primary hypogonadism | Clinical |
| 28039179 | Primary hypogonadism | Clinical |
| 30936376 | Primary hypogonadism | Clinical |
| 32556068 | Primary hypogonadism | Clinical |
| 33613847 | Primary hypogonadism | Clinical |
| 34062486 | Primary hypogonadism  & sexual life | Clinical |
| 33646368 | Secondary hypogonadism | Clinical |
| 24610577 | Secondary hypogonadism | Clinical |
| 32507965 | Secondary hypogonadism | Clinical |
| 30861560 | Secondary hypogonadism | Clinical |
| 31021376 | Secondary hypogonadism | Clinical |
| 31235040 | Secondary hypogonadism | Clinical |
| 31672171 | Libido & sexual life | Clinical |
| 17911606 | Pregnancy | Preclinical |
| 19811462 | Pregnancy | Preclinical |
| 21949023 | Pregnancy | Preclinical |
| 23261407 | Pregnancy | Preclinical |
| 27362903 | Pregnancy | Preclinical |
| 30241195 | Pregnancy | Clinical |
| 30262396 | Pregnancy | Review |
| 30262400 | Pregnancy | Clinical |
| 30454709 | Pregnancy | Clinical |
| 33768686 | Pregnancy | Clinical |
| 27062127 | Pregnancy | Review |
| 30730328 | Pregnancy | Clinical |
| 32073510 | Pregnancy | Clinical |
| 23782245 | Pregnancy | Preclinical |
| 26786669 | Pregnancy | Preclinical |
| 30500923 | Pregnancy | Preclinical |
| 30833943 | Pregnancy | Preclinical |
| 31138782 | Pregnancy | Preclinical |
| 32051396 | Pregnancy | Preclinical |
